# Supplementary material for: Osteoblast-intrinsic defect in glucose metabolism impairs bone formation in type II diabetic male mice
Source: eLife. 2023 May 5;12:e85714. doi: 10.7554/eLife.85714 (PMC10198725; doi:10.7554/eLife.85714)
Supplement: Figure 3—source data 1. [file elife-85714-fig3-data1.zip › Long_20-12-2022-RA-eLife-85714R1_Figure_3_Source_data_1.pdf]

Figure 3F Raw data

1 2 3 4 5 6 7 8

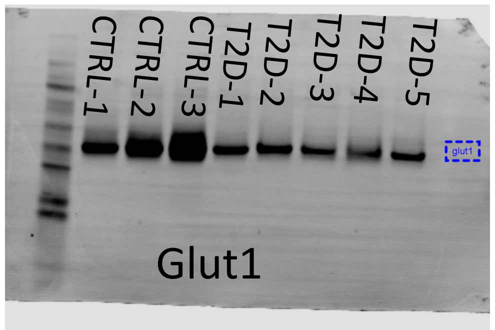

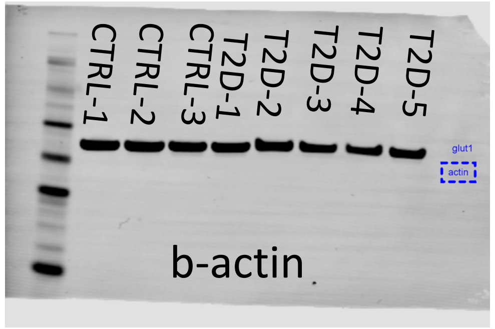

Lanes 1-6 used in figure

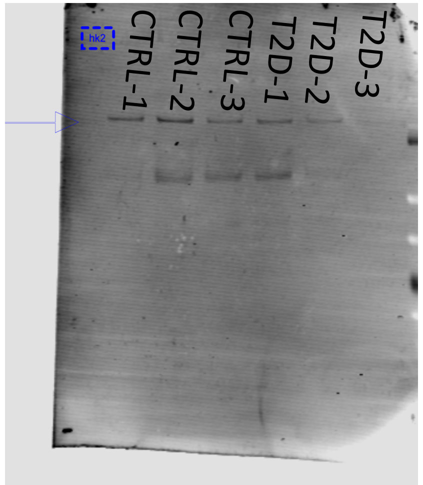

HK2

Pfkfb3 (not presented in the figure)

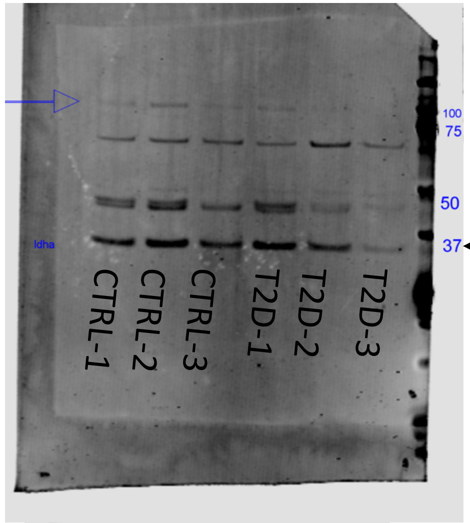

Hif1a (not presented in the figure)

LDHA

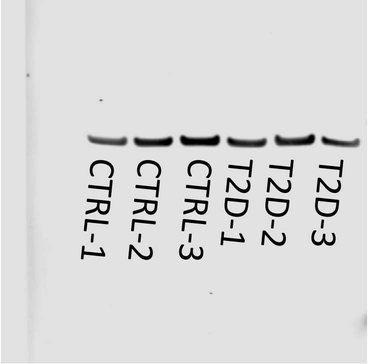

b-actin
