# Supplementary figures and images for: Osteoblast-intrinsic defect in glucose metabolism impairs bone formation in type II diabetic male mice
Source: eLife. 2023 May 5;12:e85714. doi: 10.7554/eLife.85714 (PMC10198725; doi:10.7554/eLife.85714)

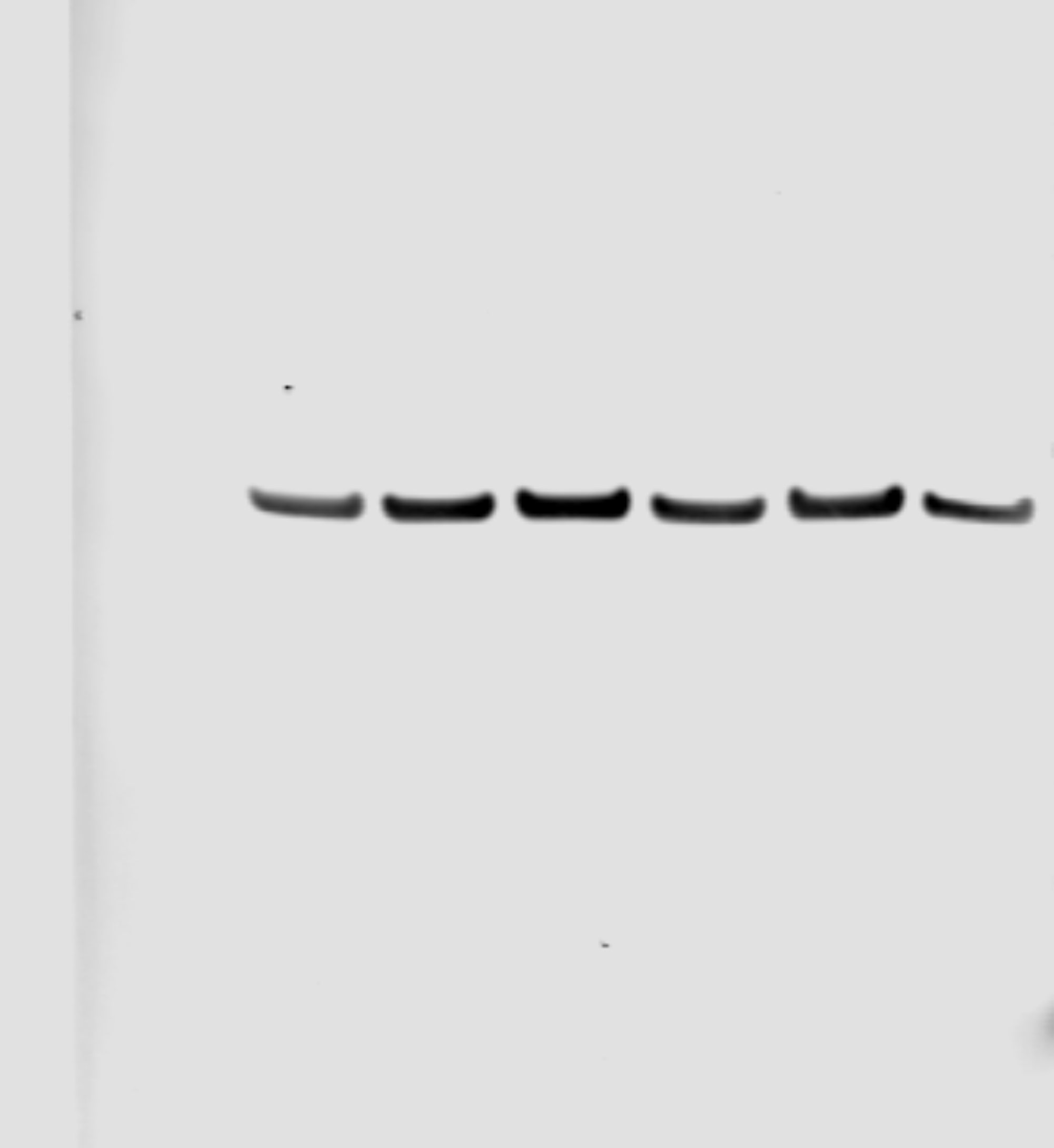

Supplement: Figure 3—source data 1. [file elife-85714-fig3-data1.zip › Long_20-12-2022-RA-eLife-85714R1_Figure_3_Source_data_5.tif]

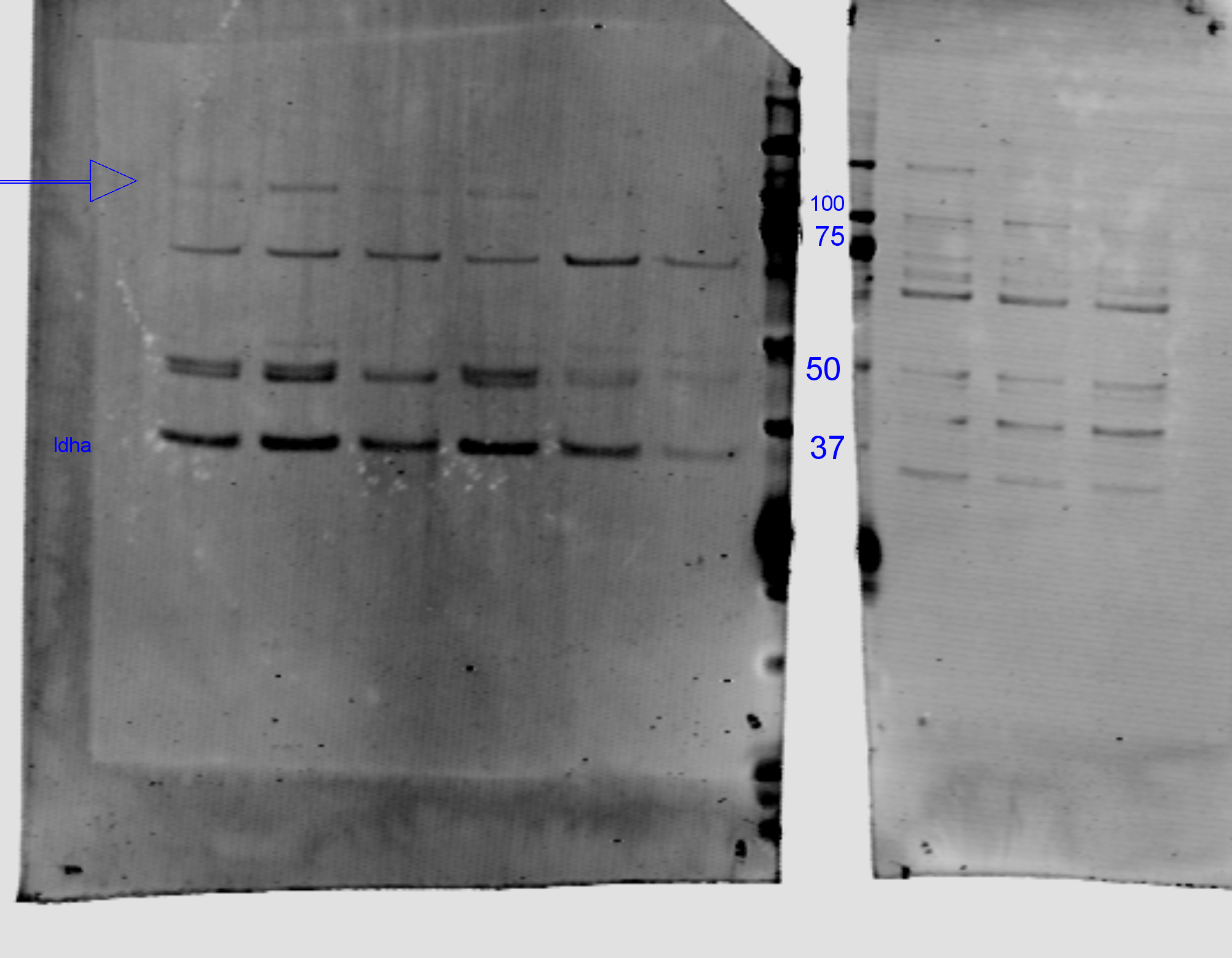

Supplement: Figure 3—source data 1. [file elife-85714-fig3-data1.zip › Long_20-12-2022-RA-eLife-85714R1_Figure_3_Source_data_4.tif]

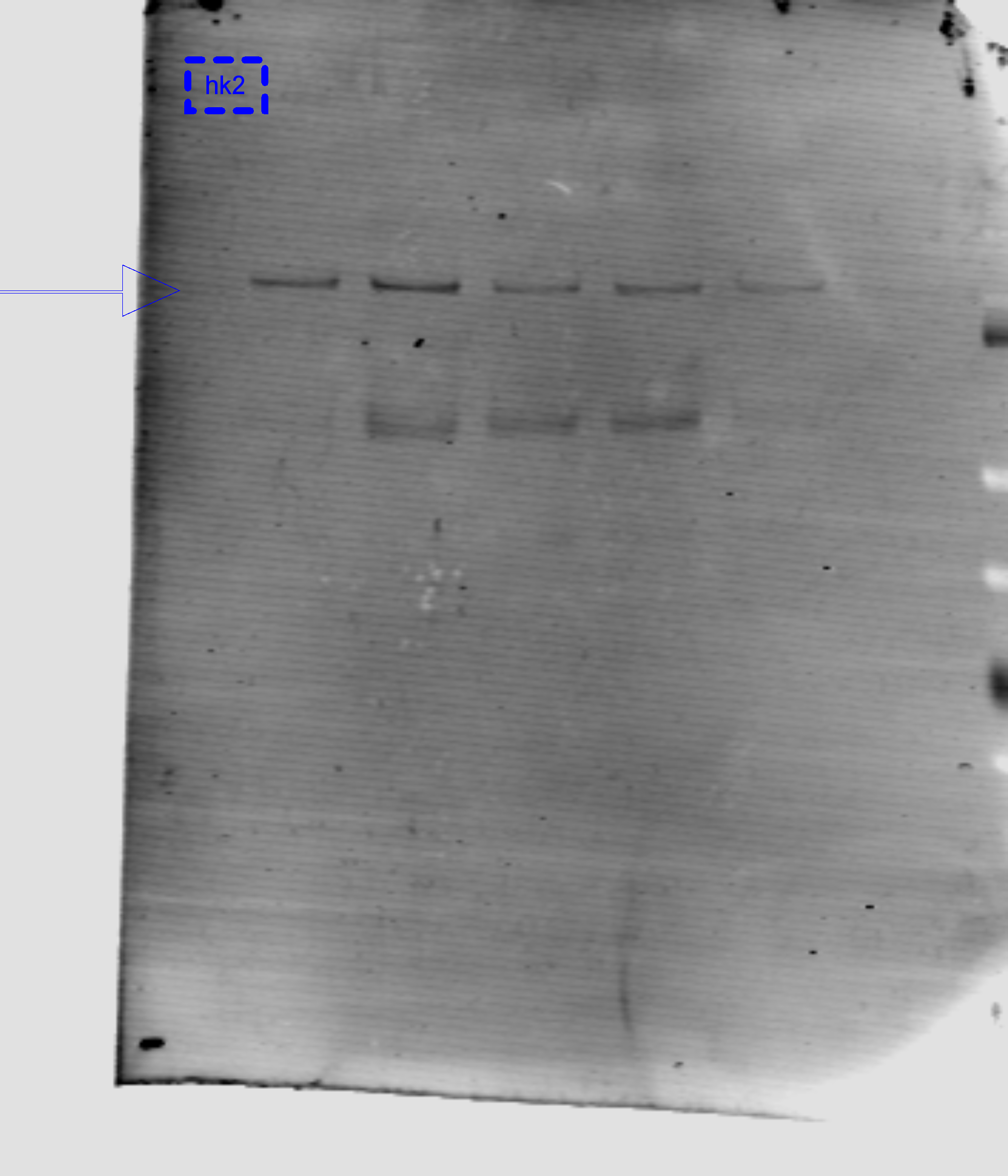

Supplement: Figure 3—source data 1. [file elife-85714-fig3-data1.zip › Long_20-12-2022-RA-eLife-85714R1_Figure_3_Source_data_3.tif]

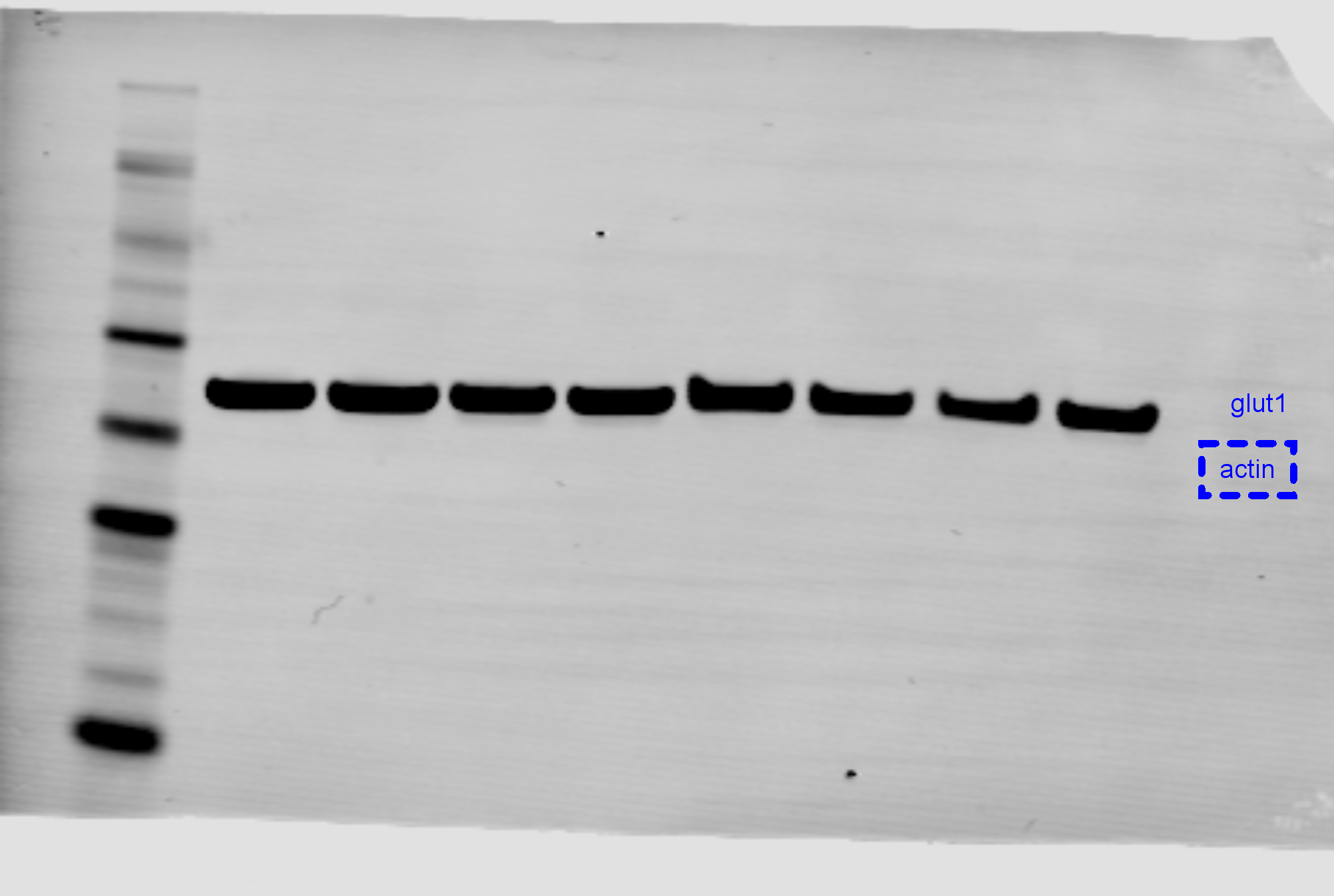

Supplement: Figure 3—source data 1. [file elife-85714-fig3-data1.zip › Long_20-12-2022-RA-eLife-85714R1_Figure_3_Source_data_2.tif]

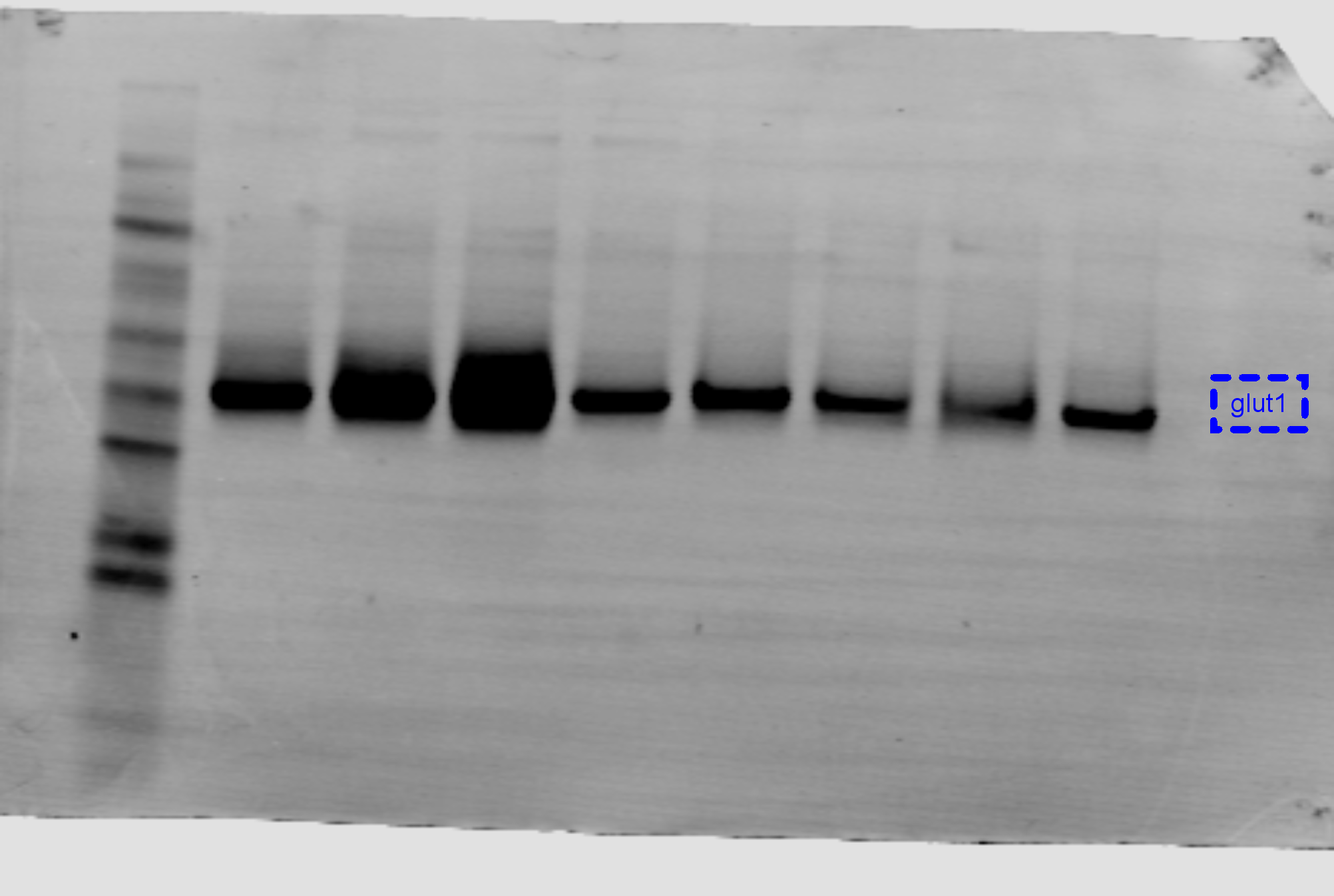

Supplement: Figure 3—source data 1. [file elife-85714-fig3-data1.zip › Long_20-12-2022-RA-eLife-85714R1_Figure_3_Source_data_1.tif]

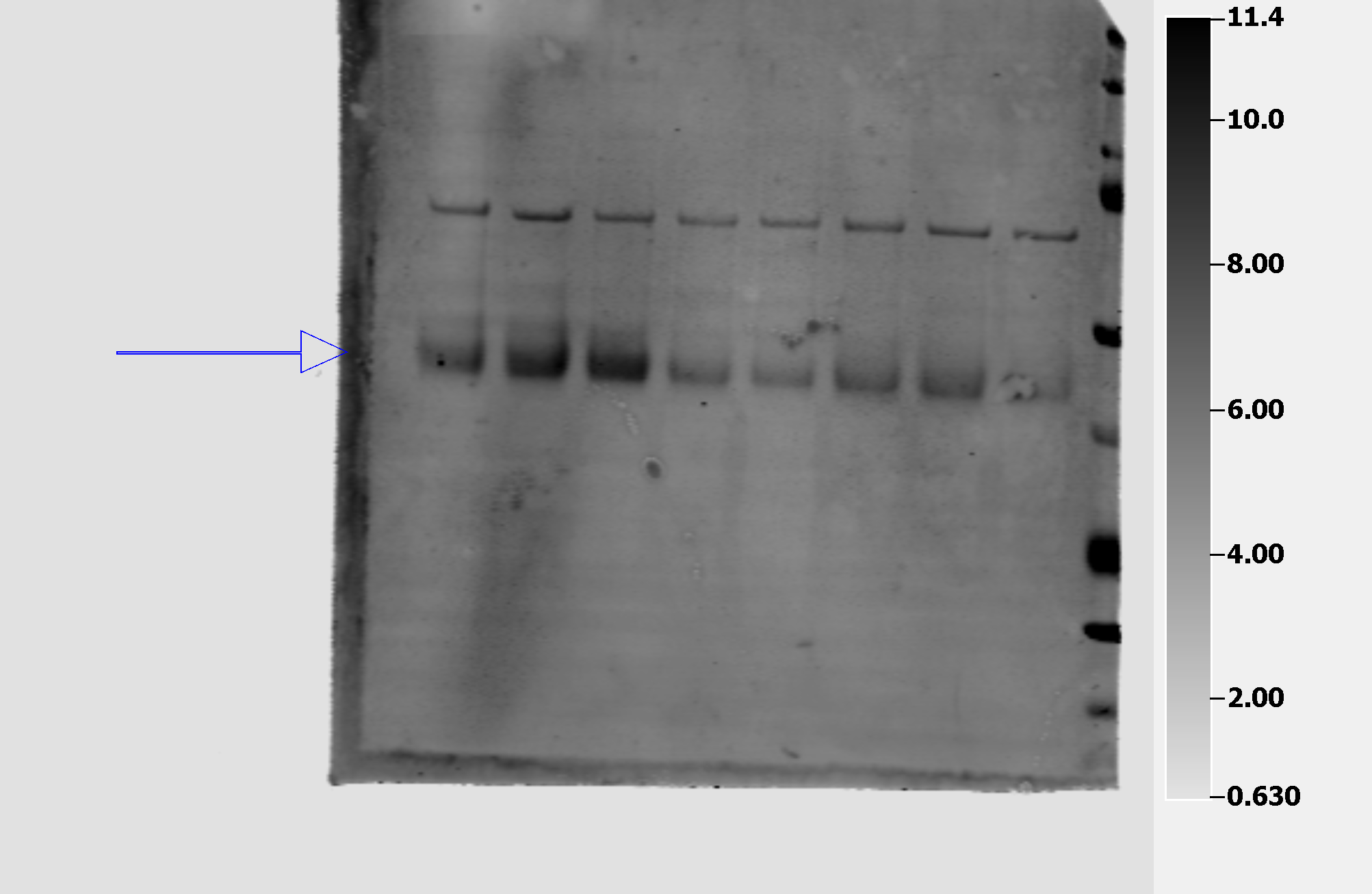

Supplement: Figure 7—figure supplement 2—source data 1. [file elife-85714-fig7-figsupp2-data1.zip › Long_20-12-2022-RA-eLife-85714R1_Figure_7_supplement_figure_2_source_data_1.tif]

Figure S8A raw data

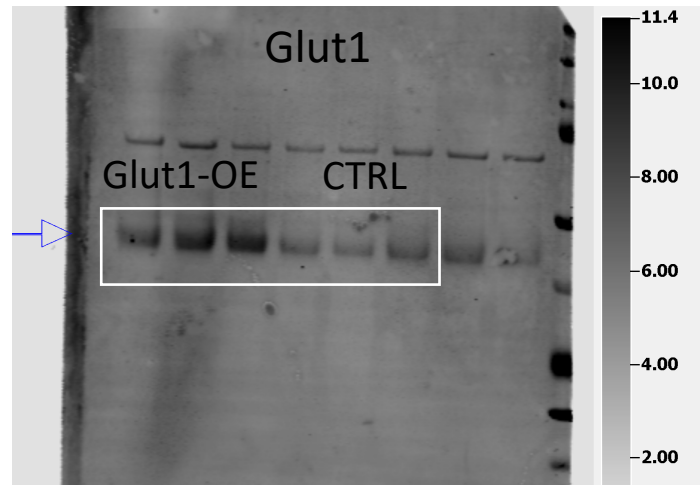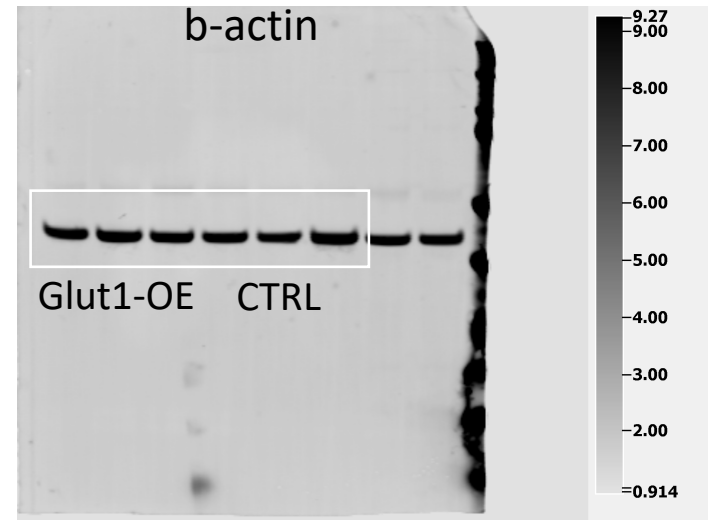

Supplement: Figure 7—figure supplement 2—source data 2. [file elife-85714-fig7-figsupp2-data2.zip › Long_20-12-2022-RA-eLife-85714R1_Figure_7_supplement_figure_2_source_data_1.pdf]

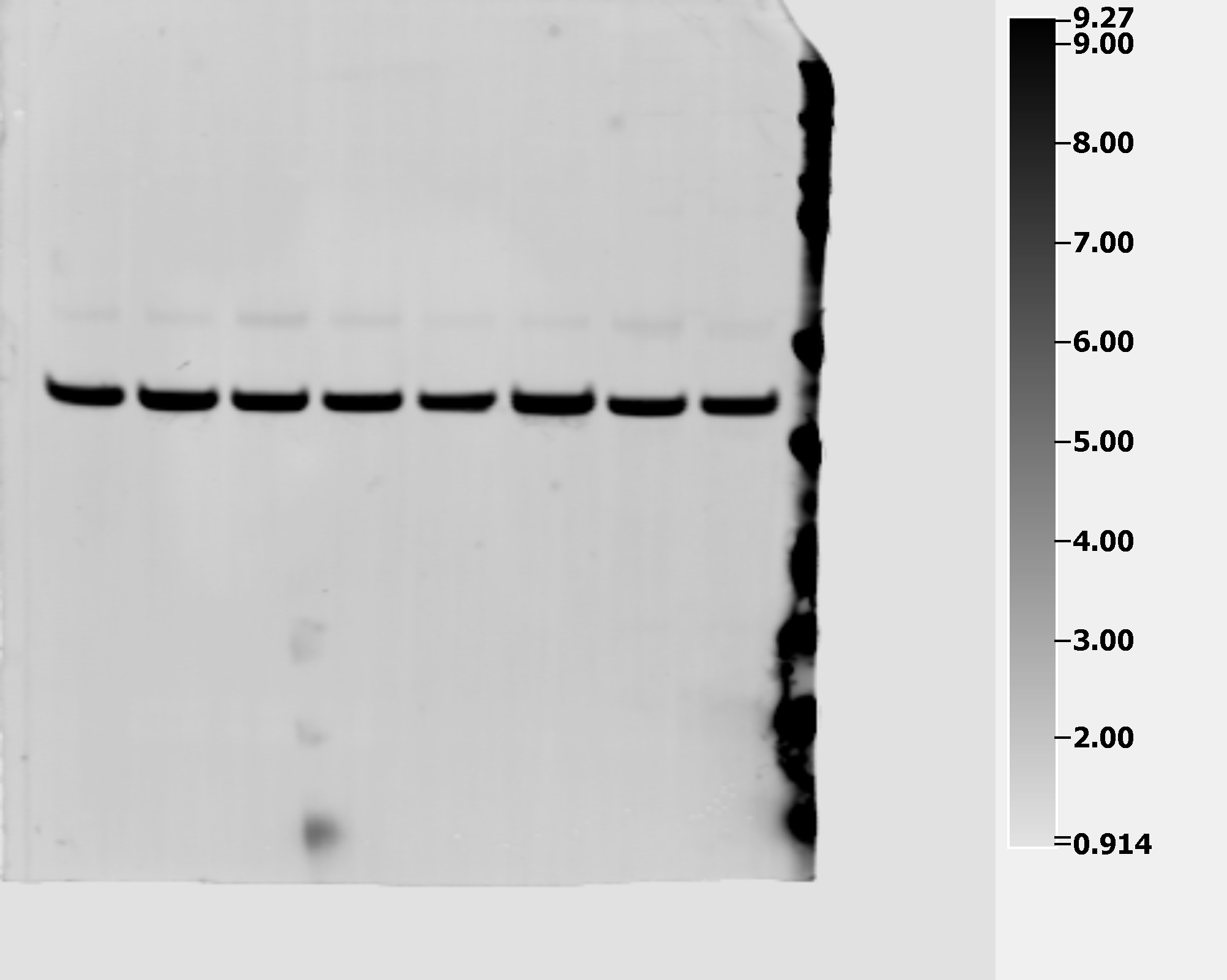

Supplement: Figure 7—figure supplement 2—source data 2. [file elife-85714-fig7-figsupp2-data2.zip › Long_20-12-2022-RA-eLife-85714R1_Figure_7_supplement_figure2_source_data_2.tif]
