# Supplementary material for: Osteoblast-intrinsic defect in glucose metabolism impairs bone formation in type II diabetic male mice
Source: eLife. 2023 May 5;12:e85714. doi: 10.7554/eLife.85714 (PMC10198725; doi:10.7554/eLife.85714)
Supplement: Figure 4—source data 1. [file elife-85714-fig4-data1.docx]

| pathway | FDR q-val | NES | cluster |
| --- | --- | --- | --- |
| HALLMARK_HYPOXIA | 0.14784402 | 1.2929997 | 0 |
| HALLMARK_OXIDATIVE_PHOSPHORYLATION | 0.15188158 | 1.309377 | 0 |
| HALLMARK_OXIDATIVE_PHOSPHORYLATION | 0 | -1.7547221 | 1 |
| HALLMARK_OXIDATIVE_PHOSPHORYLATION | 0.006597281 | 1.4519761 | 3 |
| HALLMARK_GLYCOLYSIS | 0.016376715 | 1.4558023 | 3 |
| HALLMARK_MTORC1_SIGNALING | 0.027157664 | 1.4052365 | 3 |
| HALLMARK_UNFOLDED_PROTEIN_RESPONSE | 0.030373076 | 1.3959063 | 3 |
| HALLMARK_OXIDATIVE_PHOSPHORYLATION | 0 | -1.786996 | 5 |
| HALLMARK_REACTIVE_OXYGEN_SPECIES_PATHWAY | 0.007653012 | -1.5777211 | 5 |
| HALLMARK_GLYCOLYSIS | 0.13431813 | -1.3768505 | 5 |
| HALLMARK_FATTY_ACID_METABOLISM | 0.20335594 | -1.3210925 | 5 |
| HALLMARK_CHOLESTEROL_HOMEOSTASIS | 0.20843688 | -1.3005534 | 5 |
| HALLMARK_MTORC1_SIGNALING | 0.19785942 | 1.3055938 | 7 |
| HALLMARK_UNFOLDED_PROTEIN_RESPONSE | 0.20464511 | 1.2876589 | 7 |
| HALLMARK_OXIDATIVE_PHOSPHORYLATION | 0.09714227 | -1.5019742 | 15 |

Figure 4-source data 1
