# Supplementary material for: Osteoblast-intrinsic defect in glucose metabolism impairs bone formation in type II diabetic male mice
Source: eLife. 2023 May 5;12:e85714. doi: 10.7554/eLife.85714 (PMC10198725; doi:10.7554/eLife.85714)
Supplement: Supplementary file 1. [file elife-85714-supp1.docx]

| **Supplemental File 1. GSEA analysis of each cluster (T2D VS CTRL, FDR q<0.25)** | | | | |
| --- | --- | --- | --- | --- |
| **cluster 0** | **NES** | **NOM p-val** | **FDR q-val** | **FWER p-val** |
| HALLMARK_MYC_TARGETS_V1 | 1.40818 | 0.00000 | 0.03393 | 0.03300 |
| HALLMARK_TGF_BETA_SIGNALING | 1.34864 | 0.01378 | 0.11234 | 0.19100 |
| HALLMARK_MITOTIC_SPINDLE | 1.30769 | 0.00307 | 0.12674 | 0.45600 |
| HALLMARK_APICAL_JUNCTION | 1.32803 | 0.00000 | 0.13288 | 0.31600 |
| HALLMARK_HYPOXIA | 1.29300 | 0.00613 | 0.14784 | 0.56300 |
| HALLMARK_OXIDATIVE_PHOSPHORYLATION | 1.30938 | 0.00000 | 0.15188 | 0.44200 |
| HALLMARK_PI3K_AKT_MTOR_SIGNALING | 1.27124 | 0.04198 | 0.19935 | 0.73500 |
| **cluster 1** | **NES** | **NOM p-val** | **FDR q-val** | **FWER p-val** |
| HALLMARK_OXIDATIVE_PHOSPHORYLATION | -1.75472 | 0.00000 | 0.00000 | 0.00000 |
| HALLMARK_PANCREAS_BETA_CELLS | -1.57255 | 0.00440 | 0.02150 | 0.03400 |
| HALLMARK_DNA_REPAIR | -1.40689 | 0.01965 | 0.17376 | 0.34600 |
| HALLMARK_COAGULATION | -1.36852 | 0.02731 | 0.19355 | 0.48400 |
| HALLMARK_MITOTIC_SPINDLE | 1.68012 | 0.00000 | 0.00000 | 0.00000 |
| **cluster 2** | **NES** | **NOM p-val** | **FDR q-val** | **FWER p-val** |
| HALLMARK_INTERFERON_ALPHA_RESPONSE | 1.42236 | 0.00111 | 0.02540 | 0.02400 |
| HALLMARK_INTERFERON_GAMMA_RESPONSE | 1.38382 | 0.00000 | 0.04394 | 0.08400 |
| HALLMARK_MITOTIC_SPINDLE | 1.36879 | 0.00000 | 0.05131 | 0.14200 |
| HALLMARK_UV_RESPONSE_DN | 1.29696 | 0.02119 | 0.24970 | 0.62800 |
| **cluster 3** | **NES** | **NOM p-val** | **FDR q-val** | **FWER p-val** |
| HALLMARK_TNFA_SIGNALING_VIA_NFKB | -1.55775 | 0.00000 | 0.04143 | 0.02300 |
| HALLMARK_OXIDATIVE_PHOSPHORYLATION | 1.45198 | 0.00000 | 0.00660 | 0.02300 |
| HALLMARK_MYC_TARGETS_V1 | 1.45560 | 0.00000 | 0.00819 | 0.01900 |
| HALLMARK_GLYCOLYSIS | 1.45580 | 0.00000 | 0.01638 | 0.01900 |
| HALLMARK_MTORC1_SIGNALING | 1.40524 | 0.00000 | 0.02716 | 0.11900 |
| HALLMARK_UNFOLDED_PROTEIN_RESPONSE | 1.39591 | 0.00578 | 0.03037 | 0.16200 |
| HALLMARK_PROTEIN_SECRETION | 1.38763 | 0.00828 | 0.03176 | 0.19800 |
| HALLMARK_MYC_TARGETS_V2 | 1.33809 | 0.03511 | 0.09300 | 0.52700 |
| HALLMARK_PI3K_AKT_MTOR_SIGNALING | 1.33149 | 0.02959 | 0.09318 | 0.57800 |
| HALLMARK_EPITHELIAL_MESENCHYMAL_TRANSITION | 1.28440 | 0.03548 | 0.19707 | 0.88000 |
| **cluster 5** | **NES** | **NOM p-val** | **FDR q-val** | **FWER p-val** |
| HALLMARK_OXIDATIVE_PHOSPHORYLATION | -1.78700 | 0.00000 | 0.00000 | 0.00000 |
| HALLMARK_REACTIVE_OXYGEN_SPECIES_PATHWAY | -1.57772 | 0.00000 | 0.00765 | 0.01200 |
| HALLMARK_DNA_REPAIR | -1.48236 | 0.00289 | 0.03545 | 0.12800 |
| HALLMARK_MYC_TARGETS_V1 | -1.50350 | 0.00140 | 0.03663 | 0.08200 |
| HALLMARK_PANCREAS_BETA_CELLS | -1.48667 | 0.01621 | 0.04252 | 0.12300 |
| HALLMARK_COAGULATION | -1.42147 | 0.01302 | 0.08430 | 0.32800 |
| HALLMARK_GLYCOLYSIS | -1.37685 | 0.00536 | 0.13432 | 0.53400 |
| HALLMARK_ALLOGRAFT_REJECTION | -1.35400 | 0.01335 | 0.15978 | 0.64900 |
| HALLMARK_UV_RESPONSE_UP | -1.31752 | 0.03890 | 0.18925 | 0.78000 |
| HALLMARK_FATTY_ACID_METABOLISM | -1.32109 | 0.03545 | 0.20336 | 0.77400 |
| HALLMARK_CHOLESTEROL_HOMEOSTASIS | -1.30055 | 0.08244 | 0.20844 | 0.84000 |
| HALLMARK_ADIPOGENESIS | -1.26284 | 0.04237 | 0.23993 | 0.93200 |
| HALLMARK_XENOBIOTIC_METABOLISM | -1.27836 | 0.05355 | 0.24202 | 0.89600 |
| HALLMARK_P53_PATHWAY | -1.26704 | 0.06334 | 0.24694 | 0.92100 |
| HALLMARK_MITOTIC_SPINDLE | 1.43680 | 0.00380 | 0.08551 | 0.19900 |
| HALLMARK_UV_RESPONSE_DN | 1.26771 | 0.04777 | 0.21642 | 0.69300 |
| **cluster 7** | **NES** | **NOM p-val** | **FDR q-val** | **FWER p-val** |
| HALLMARK_PI3K_AKT_MTOR_SIGNALING | 1.30056 | 0.03348 | 0.18681 | 0.76700 |
| HALLMARK_MTORC1_SIGNALING | 1.30559 | 0.01486 | 0.19786 | 0.74400 |
| HALLMARK_INTERFERON_GAMMA_RESPONSE | 1.31373 | 0.01049 | 0.20222 | 0.69300 |
| HALLMARK_UNFOLDED_PROTEIN_RESPONSE | 1.28766 | 0.03889 | 0.20465 | 0.83000 |
| HALLMARK_ANDROGEN_RESPONSE | 1.26514 | 0.06208 | 0.23163 | 0.91400 |
| HALLMARK_INTERFERON_ALPHA_RESPONSE | 1.31479 | 0.03837 | 0.23848 | 0.68800 |
| HALLMARK_MITOTIC_SPINDLE | 1.26936 | 0.03347 | 0.23980 | 0.90000 |
| HALLMARK_PANCREAS_BETA_CELLS | -1.40230 | 0.05911 | 0.09226 | 0.09700 |
| **cluster 15** | **NES** | **NOM p-val** | **FDR q-val** | **FWER p-val** |
| HALLMARK_OXIDATIVE_PHOSPHORYLATION | -0.64650 | -1.50197 | 0.00000 | 0.09714 |
| **cluster 18** | **NES** | **NOM p-val** | **FDR q-val** | **FWER p-val** |
| HALLMARK_EPITHELIAL_MESENCHYMAL_TRANSITION | 1.47963 | 0.00000 | 0.13895 | 0.12400 |
