# Supplementary material for: Osteoblast-intrinsic defect in glucose metabolism impairs bone formation in type II diabetic male mice
Source: eLife. 2023 May 5;12:e85714. doi: 10.7554/eLife.85714 (PMC10198725; doi:10.7554/eLife.85714)
Supplement: Supplementary file 2. [file elife-85714-supp2.docx]

**Supplemental File 2. Sequences of Primers**

| Primers | Forward | Reverse |
| --- | --- | --- |
| Alpl  Spp1  Bmp2  Bglap  Sp7  Runx2  Slc2a1  Slc2a3  Ldha  Pfkfb3  Ibsp  Col1a1  Hk2  Pgm1  Gapdh  Hif1a  Ldhb  Idh2  Idh3b  Ndufc1  Ndufa4  Ndufaf2  Ndufaf4  Actb  Fabp4  Pparg | ACTGATGTGGAATACGAACTGG  GCTTGGCTTATGGACTGAGGTC  AACACCGTGCGCAGCTTCCATC  CACCTAGCAGACACCATGAG  ATG GCG TCC TCT CTG CTT G  GTAGCCAGGTTCAACGATCTG  GATTGGTTCCTTCTCTGTCGG  CGCTTCTCATCTCCATTGTCC  GCTCCCCAGAACAAGATTACAG  GTGTGGCTTCCACTTCTGCT  CCGGCCACGCTACTTTCTT  ATGCCTACTTTTATCCTCCTCTG  GTCAGACTCATCCTGGTGAAG  CAACGCACTGAAGGAGCTACTC  CATCACTGCCACCCAGAAGACTG  CCTGCACTGAATCAAGAGGTTGC  CCTCAGATCGTCAAGTACAGCC  CAAGGAGTGGGAGGTGTATAAC  TCATTGAGTGCCTGAAGATCG  TCAACACGGTCGAAGTTCTATG  CCCAGCTTGATTCCTCTCTTC  ACTGGAGAGGGCAGACTATT  GTAACTGGGCTCGTCCATATTC  GTGACGTTGACATCCGTAAAGA  GAAGCTTGTCTCCAGTGAAAAC  GGAAAGACAACGGACAAATCAC | AGTTCAGTGCGGTTCCAG  CCTTAGACTCACCGCTCTTCATG  CGGAAGATCTGGAGTTCTGCAG  GTTCACTACCTTATTGCCCTCC  TGA AAG GTC AGC GTA TGG CTT  CCGTCCACTGTCACTTTAATAGC  CCCAGGATCAGCATCTCAAAG  TGAAGATAGTATTGACCACGCC  TCGCCCTTGAGTTTGTCTTC  CATGTTTTGTCCGGGCAGC  GGACTGGAAACCGTTTC  TCTTCATTGTTTTCCTCTTCGTTTG  CCTTCTGAATTCCGTCCTTATCG  GGCACCAAGTTCTTCACAGAGG  ATGCCAGTGAGCTTCCCGTTCAG  CCATCAGAAGGACTTGCTGGCT  ATCCGCTTCCAATCACACGGTG  TCAAGTAGAGCGGCCATTTC  CAAGAACAAGCCATCCCCTAG  CATCTTCATTGTGTGTTTGGATGA  GGCTCTGGGTTGTTCTTTCT  GGTGGAGTCTTCCTTGTTCTTC  CATCGCTGGATTCTTCTCATCT  GCCGGACTCATCGTACTCC  GACCAAATCCCCATTTACGC  TACGGATCGAAACTGGCAC |
